# HER4 in breast cancer: comparison of antibodies against intra- and extra-cellular domains of HER4

**DOI:** 10.1186/bcr1394

**Published:** 2006-04-07

**Authors:** Sian M Tovey, Barbara Dunne, Caroline J Witton, Timothy G Cooke, John MS Bartlett

**Affiliations:** 1 Endocrine Cancer Group, Section of Surgical and Translational Research, Glasgow University, UK; 2 Department of Pathology, Glasgow Royal Infirmary, Glasgow, UK

## Abstract

**Introduction:**

We have previously linked HER4 expression with increased survival in breast cancer. However, other reports have associated HER4 with adverse prognostic significance. One possible explanation for the conflicting reports may be that these results are antibody dependent. The HER4 protein is enzymatically cleaved, which may alter the function of its intracellular domain (ICD). We have therefore compared the staining patterns of antibodies against its intracellular and extracellular domains using tissue microarray technology.

**Methods:**

Immunohistochemistry was performed and evaluated on tumours from 402 tamoxifen treated oestrogen receptor positive patients. The HFR1 antibody recognises the ICD of HER4 and thus recognises both the intact receptor and the cleaved ICD. The H4.77.16 clone recognises an extracellular domain of HER4 and thus detects the full length receptor only.

**Results:**

Both antibodies demonstrated nuclear, cytoplasmic and membranous staining. Concordance between the membrane staining patterns was high (88.44%, kappa 0.426). The HFR1 antibody, however, demonstrated generally higher levels of cytoplasmic staining (concordance 74.77%, kappa 0.351). The antibodies demonstrated very different patterns of nuclear staining. Over 60% of patients stained with the H4.77.16 had no nuclear staining whereas the vast majority showed staining with the HFR1 antibody (concordance 40.12%, kappa 0.051). Neither antibody demonstrated relationships between membranous or cytoplasmic HER4 staining and survival, although associations were seen with known poor prognostic markers. Cases with H4.77.16-determined nuclear staining had significantly poorer survival outcomes.

**Conclusion:**

The difference in antigen site may explain the different staining patterns we have seen with respect to location; with each antibody appearing to select for distinct compartments. Thus, HFR1 may select for cytoplasmic and nuclear HER4 ICD, whilst H4.77.16 selects for membranous HER4 and/or HER4 being recycled in cytoplasm or nucleus. This ability to distinguish between site and function of HER4 and its fragments is particularly important, with recent evidence highlighting the different functions of nuclear and mitochondrial HER4.

## Introduction

Overexpression of members of the human epidermal growth factor receptor (HER) family has been widely studied in breast cancer. Whereas the biology underlying the role of HER2 and epidermal growth factor receptor (EGFR) has been increasingly documented, more confusion exists in establishing a role for HER4 (c-erbB-4). We have shown that, in contrast to other HER family members, HER4 expression is associated with increased survival and lower proliferation indices [1,2]. These results are supported by data linking HER4 to established good prognostic indicators such as a lower grade of tumour [3,4], oestrogen receptor (ER) positivity [5] and low proliferation indices [6]. However, whilst other groups have also demonstrated a link between HER4 positivity and a longer disease free interval [7], conflicting reports have associated HER4 with an adverse prognostic significance [8]. More recently, evidence from a large series of patients has suggested that the prognostic value of HER4 overexpression is dependent on coexpression with other HER family members [9]. In this study, when the group was looked at as a whole, HER4 status was not related to survival [9]. In cases showing expression of one family member only (homodimers), however, they found a significant association between HER4 homodimer-expressing tumours and improved disease free survival.

There are intrinsic problems in comparing these studies and their outcomes. Different cut off points for positivity have been chosen depending on the study and the modality of staining looked at (membrane, cytoplasm and nuclear). Some groups have reported staining in all three locations, whilst others have found no membranous [8] or no nuclear staining [7]. Three different antibodies have been used in these studies. The HFR1 clone developed by the Gullick group has been the most widely used [3,4,8-10]. This group demonstrated the ability of this antibody to recognise HER4 by immunoprecipitation, western blotting and immuno-staining of NH3T3 cells transfected with HER4. They demonstrated no cross-reactivity with EGFR using A431 cell lysates or with HER3 or HER4 using lysates from SKBR3 or 293/HER3 cells. A Santa Cruz antibody C18 has also been used by one group [7]. In our previously study on frozen tissue, we used a Neomarkers antibody H4.77.16 [1].

Recent studies have substantially enhanced our understanding of the many functions of HER4. Indeed, as well as acting as a membrane signal transduction receptor, nuclear HER4 is required for mammary gland development and lactation through gene regulation in conjunction with STAT5A [11,12], and mitochondrial HER4 has been shown to mediate apoptosis in the mitochondria via BAK [13].

Recent evidence suggests that, as with HER2 and EGFR, the HER4 protein can be enzymatically cleaved, which may markedly alter the function of the intracellular domain of the receptor. Cleavage occurs within the juxtamembrane region through the activity of tumour necrosis factor-α-converting enzyme (TACE) followed by further proteolysis processing by presenilin-dependent γ-secretase activity [14,15] to release the cleaved intracellular domain (4ICD). Indeed, this 4ICD has been shown to harbour a BCL-2 homology 3 (BH3) domain and can independently function as a BH3-only protein (pro-apoptotic members of the BCL-2 family required to initiate mitochondria dysfunction), so mediating cellular apoptosis [13]. However, it has also been demonstrated in the nucleus acting as a chaperone for STAT5A [12] to alter gene regulation. Thus, it is essential to determine both the location and intensity of staining for HER4 in order to fully understand its function *in vivo*. The variance in reported results for *in vivo *analysis of HER4 expression may be a reflection of differing antibody specificities, especially with respect to the intracellular and extracellular domains of the protein. Indeed, one recent study using the HFR1 antibody demonstrated very different correlations in terms of survival depending on cellular location of HER4 staining. Whilst membranous HER4 was associated with a good prognostic outlook, nuclear HER4 was associated with significantly shorter survival times [16].

Thus, one possible explanation for the conflicting reports on HER4 and its association with survival may be that the results are antibody dependent. HFR1 recognises the intracellular domain of HER4 and is thus able to recognise both the intact receptor and the cleaved ICD as it traffics through the cell. However, the H4.77.16 clone recognises an extracellular domain of HER4 and thus, on tissue sections, detects the full length receptor, not the cleaved ICD. We have therefore compared the staining patterns of these most widely used antibodies in standardised conditions in a large cohort of patients using tissue microarray technology.

## Materials and methods

### Patients

The local ethics committee granted ethical approval for the study. Four hundred and two, tamoxifen treated, ER positive patients, were selected from a database of sequentially diagnosed patients presenting with operable breast cancer between 1980 and 1999 for a retrospective study relating molecular markers to tamoxifen resistance [17]. They had standard adjuvant treatment according to protocols at the time of diagnosis.

### Tissue microarray construction

Three 0.6 mm^2 ^cores of breast cancer tissue were removed from representative tumour areas on each block identified by a pathologist (BD). These cores were used to construct recipient array blocks in triplicate (80 to 120 cores per block). Cores of normal skin, smooth muscle, testes, lymph node, placenta and tonsil were also included in the tissue microarray (TMA) as controls. If one or more core was uninformative because of loss or lack of tumour in core then the scoring results were taken from the remaining core(s).

### Immunohistochemistry

Sections were de-waxed and hydrated then endogenous peroxide was blocked with hydrogen peroxide. There was no antigen retrieval required. Sections were incubated in serum free block (DAKO UK Ltd, Ely, Cambridgeshire, UK) to block non-specific background staining then endogenous biotin was blocked with an Avidin/Biotin blocking agent (Vector Laboratories, Peterborough, UK). Incubation with the primary antibody diluted in a tris-HCL buffer (DAKO) was performed at room temperature. The HFR1 antibody was used at a concentration of 4 μg/ml for 2 hours, and the H4.77.16 antibody was used at a concentration of 50 μg/ml for 2 hours, both at room temperature. The DAKO LSAB+ kit was used for signal amplification. Washes, between all steps, were performed with TBS solution (Tris saline buffer, pH 7.6). Detection was then completed with incubation with a 3,3'diaminobenzidine (DAB) solution (Vector) diluted in dH_2_0 for 10 minutes. Finally the sections were counterstained, dehydrated and mounted. A control slide was incubated in each run with an isotype matched control antibody to ensure no false positive staining. All slides for each antibody were stained in a single staining run to minimise batch to batch variation.

Staining and scoring for HER1, HER2 and HER3 was performed for the same set of patients as described previously [17].

### Scoring

One scorer scored all cases having previously double scored a series of TMA slides (including the Herceptest and ER slides) with an experienced diagnostic scorer achieving an ICCC (intra-class correlation coefficient) of 0.94 (*n *= 890) for membrane staining and 0.84 (*n *= 827) for nuclear staining.

Membrane staining was scored using the Herceptest method. Cores with over 10% of strong membrane staining were assigned 3+; cores with over 10% moderate staining were assigned 2+; and cores with over 10% of cells with incomplete membrane staining were assigned 1+. When there was any discrepancy between cores, the mean percentages stained at each intensity level were calculated. Thus, an average of at least 10% of cells with strong membranous staining over analysed cores would be required for the combined score to reach 3+. Patients were considered positive for membranous HER4 if they had at least 2+ staining intensity (for instance, at least 10% of tumour cells were scored as being moderately positive).

Nuclear and cytoplasmic scoring was performed using the Histoscore method. This involves giving a weighted score for percentages of staining seen, where the percentage of cells stained (0% to 100%) is multiplied by the staining intensity (1, 2 or 3) to give a maximum histoscore of 300. The histoscores for each core were then averaged. Patients were considered positive for cytoplasmic or nuclear HER4 if the average histoscore for the respective modality was greater than 10.

### Statistical analysis

The statistical software package SPSS version 9.0 (SPSS Inc., Chicago, IL) was used for all analysis. The Kaplan Meier life table statistical analysis was undertaken for disease free and survival analysis. Concordance between two antibodies, with regard to staining at the membrane, cytoplasm and nucleus, was evaluated using chi-square kappa values, where a value of 1 indicates perfect agreement and a value of 0 indicates that agreement is no better than chance. Correlations with clinicopathological variables and other HER family members were performed using Chi-square or Fishers test.

## Results

### Patient characteristics

Clinical and pathological characteristics, including grade, nodal status, histology, size, Nottingham Prognostic Index and age are shown in Table 1. In addition to tamoxifen, 99/399 (24.8%) patients had chemotherapy (3 unknown) and 110/399 (27.57%) had radiotherapy (3 unknown). The median duration of tamoxifen therapy was 5 years. The mean follow-up duration is 6.91 years (standard deviation (SD) 3.34 years) and median 6.45 years (range 0.64 to 18.42 years).

### Missing cores

For the H4.77.16 and HFR1 antibodies there were 61/402 (15.2%) and 43/402 (10.7%) cases, respectively, with no valid cores available for analysis. This was due to a combination of either core loss or because no tumour was present within the core.

#### Comparison of antibody staining patterns: membrane, cytoplasm and nuclear

**Figure 1:**
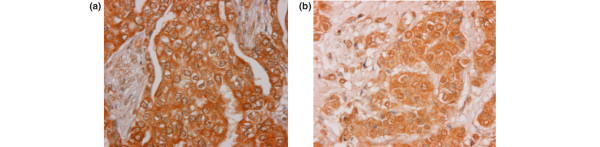
Photomicrographs of immunohistochemical staining using **(a) **H4.77.16 and **(b) **HFR1 antibodies.

**Figure 2:**
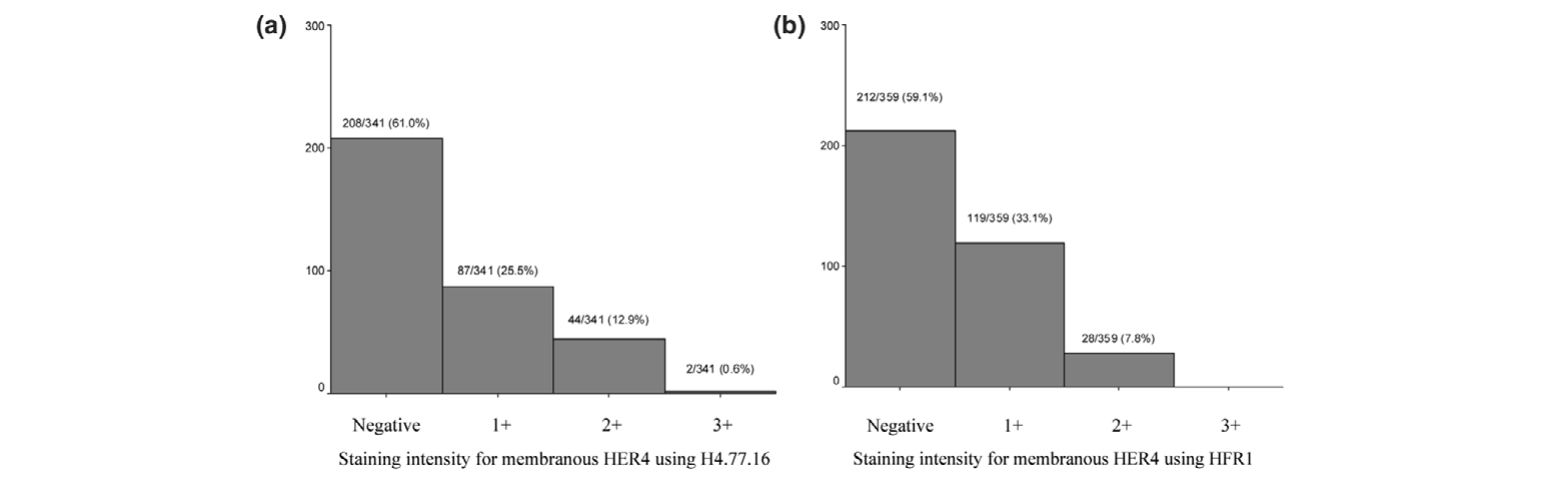
Frequency histograms for membrane staining intensity using **(a) **H4.77.16 and **(b) **HFR1.

Both antibodies demonstrated nuclear, cytoplasmic and membranous staining (Figure 1a, b). Membrane staining patterns for each antibody are shown (Figure 2a, b). Using the cutoffs previously described, 46/341 (11.4%) patients were classed as positive using the H4.77.16 antibody and 28/359 (7.0%) using the HFR1 antibody.

The concordance between membrane staining using the 2 antibodies is 88.44% (*n *= 329; Table 2) with a kappa value of 0.426 (where a value of 1 indicates perfect agreement and a value of 0 indicates that agreement is no better than chance). It can be seen in Figure 2 that the percentage of tumours that are negative is similar between the two antibodies but that the H4.77.16 antibody appears to detect a stronger intensity of staining at the membrane. This may reflect a difference in sensitivity between the antibodies or, alternatively, a difference between TACE cleaved but γ-secretase intact HER4.

**Figure 3:**
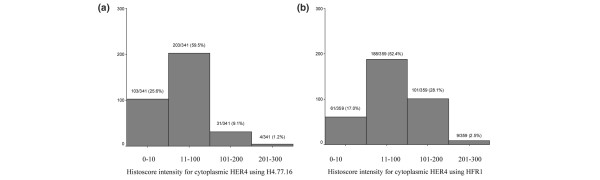
Frequency histograms for cytoplasmic staining intensity using **(a) **H4.77.16 and **(b) **HFR1.

The median cytoplasmic histoscore for H4.77.16 antibody was 36.67 (range 0 to 250) and mean 48.21 (SD 50.32). The median cytoplasmic histoscore for HFR1 was 75 (range 0 to 253) and mean 78.75 (SD 61.10). The HFR1 antibody therefore generally has higher levels of cytoplasmic staining (Figure 3a, b). Using the cut offs described previously (histoscore >10), 225/341 (66.0%) tumours were classed as positive using the H4.77.16 antibody and 293/359 (81.6%) using the HFR1 antibody. The concordance between the 2 antibody results is 74.77% (*n *= 329; Table 2) with a kappa value of 0.351. This difference may reflect the fact that HFR1 can recognise both cleaved 4ICD and the intact (recycling) HER4, whilst H4.77.16 recognises the intact form only.

**Figure 4:**
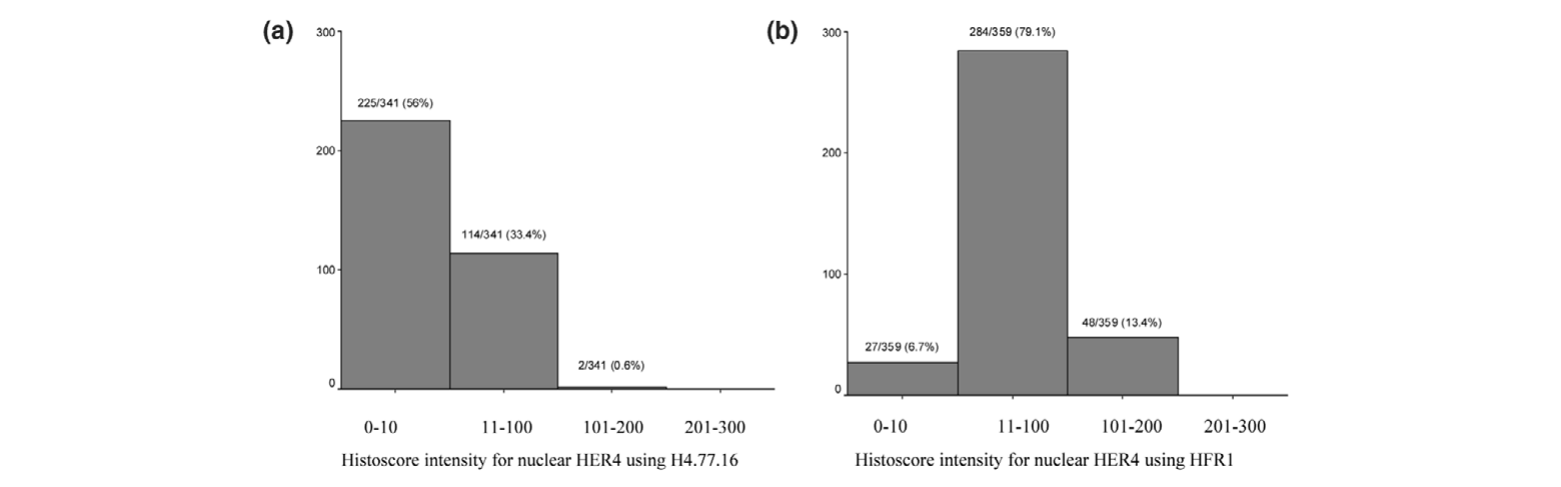
Frequency histograms for nuclear staining intensity using **(a) **H4.77.16 and **(b) **HFR1.

The median nuclear score for H4.77.16 antibody was 0 (range 0 to 200) and mean 15.01 (SD 26.42). The median nuclear score for HFR1 was 63.33 (range 0 to 200) and mean 64.83 (SD 38.65). The antibodies showed very different staining patterns in the nucleus (Figure 4a, b). Using the cut offs described previously (histoscore >10), 116/341 (34.0%) patients were classed as positive using the H4.77.16 antibody and 332/359 (89.3%) using the HFR1 antibody. Whilst over 60% of patients stained with the H4.77.16 had no nuclear staining, the vast majority showed some staining with the HFR1 antibody. Once split into positive and negative groups, the concordance between the 2 antibody results is 40.12% (*n *= 329; Table 2) with a kappa value of 0.051. This lack of agreement may well reflect the fact that the cleaved 4ICD (recognised by HFR1) is much more likely to be found in the nucleus than the intact form.

### Relationship between staining locations for each antibody

Staining patterns using the H4.77.16 antibody showed correlations between membranous staining and both cytoplasmic and nuclear staining (both *p *< 0.001, Mann Whitney). However, whilst there was a correlation between membranous and cytoplasmic staining using the HFR1 antibody (*p *= 0.008, Mann Whitney), there was no correlation between membranous and nuclear staining (*p *= 0.259, Mann Whitney).

### Relationship with clinicopathological variables and the other HER family members

We have previously reported the HER1-3 status of this cohort [17]; 6/393 (1.5%) patients are positive for HER1, 51/397 (12.8%) positive for HER2 and 56/353 (15.9%) are positive for HER3. Altogether, 98/350 (28.0%) cases are positive for one or more of HER1, HER2 or HER3.

Both antibodies demonstrated a significant relationship between membranous HER4 and increasing size and HER3 positivity (Table 3). For the patients positive for HER4 using the HFR1 antibody, 22/28 (78.57%) were also positive for another member of the HER family. For the H4.77.16 antibody, this figure was 28/46 (60.87%).

Both antibodies showed a correlation between HER4 cytoplasmic staining and increasing Nottingham Prognostic Index, nodal status, size and HER3 positivity (Table 4). Neither antibody showed any significant correlations between nuclear HER4 staining and pathological variables or HER1-3 status.

### Survival and disease free analysis

**Figure 5:**
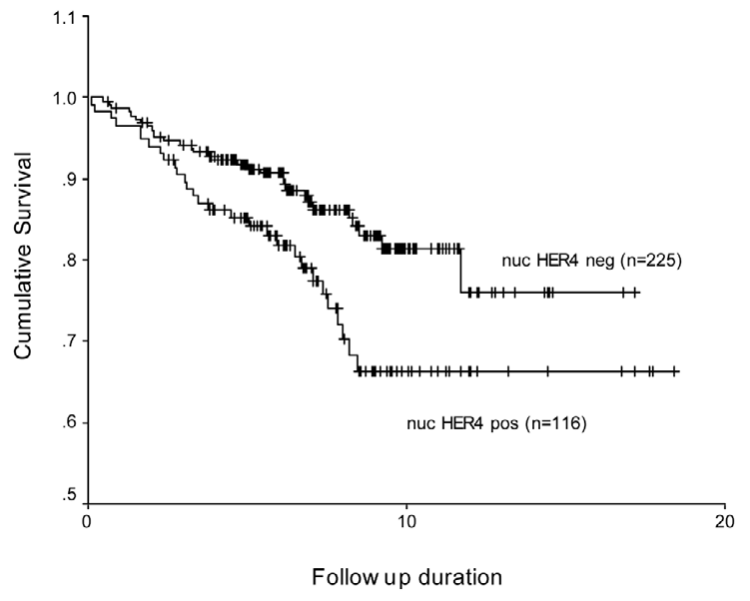
Cumulative survival differences between patients positive or negative for nuclear HER4 using the H4.77.16 antibody. Kaplan-Meier survival curves demonstrating cumulative survival differences (endpoint breast cancer related death) between patients positive or negative for nuclear (nuc) HER4 using the H4.77.16 antibody. *P *values represent log rank testing of the difference in cumulative disease free survival.

For both antibodies there was no relationship between disease free or overall survival and membranous HER4 staining. Cases who were positive for HER4 only (and not for any other members of the HER family) were identified (*n *= 6 for HFR1 and *n *= 18 for H4.77.16) but these patients again did not have significantly different rates of survival. Cytoplasmic staining was not correlated with disease free or overall survival using either antibody. However, cases demonstrating nuclear HER4 staining using the H4.77.16 antibody were significantly more likely to have poorer overall survival (*p *= 0.0124, Figure 5). There was no such correlation with survival with the HFR1 antibody.

## Discussion

We have demonstrated for the first time that the H4.77.16 antibody can be used successfully in formalin fixed tissue. In keeping with previous reports we also have found membranous, cytoplasmic and nuclear staining. The HFR-1 antibody is raised against an intracellular epitope aa1249-1264 (therefore, it will detect both the intact and cleaved form of 4ICD) whilst the H4.77.16 antibody is raised against an extracellular fragment (hence will detect only the full length HER4 protein or the cleaved extracellular domain at the cell surface). This difference in antigen site may explain the different staining patterns we have seen in terms of location, with each antibody appearing to select for distinct compartments. Thus, HFR1 may select for cytoplasmic and nuclear HER4 ICD whilst H4.77.16 selects for membranous HER4 and possibly also HER4 being recycled in cytoplasm or nucleus. This ability to distinguish between site and function of HER4 and its fragments is particularly important with recent evidence highlighting the different functions of nuclear and mitochondrial HER4. We now know that whilst HER4 at the membrane is accountable for signal transduction, mitochondrial HER4 ICD appears to be involved in apoptosis mediation [13] and nuclear HER4 is required for mammary gland development and lactation [11].

Despite the differences seen in staining location, we demonstrate that in terms of relationships with pathological variables, HER family members and prognostic importance, when tested under standardised conditions on the same set of tumours both antibodies provide generally similar results. The exception to this is the association that H4.77.26-detected nuclear HER4 has with poorer survival. This correlates with recently published results demonstrating that whilst membranous HER4 was associated with a good prognostic outlook, nuclear HER4 was associated with significantly shorter survival times [16]. Interestingly, this study also used the H4.77.16 antibody. Clearly this demonstrates that despite strong evidence for the role of the cleaved 4ICD, intact HER4 may also have a significant nuclear function.

We have not demonstrated any association between membranous HER4 and survival when considered alongside other HER family members or alone. However, we did demonstrate an association between membranous and cytoplasmic HER4 and known poor prognostic variables. Few cases in this series expressed HER4 in isolation and it is increasingly apparent that co-expression of HER4 with other HER proteins may abrogate its protective effects [9]. HER4 appears, therefore, to be protective if homodimerisation occurs, but this effect is lost if other HER members are activated by heterodimerisation with HER4. This is also consistent with data from cell lines showing that whilst HER4 can induce growth arrest and differentiation [18,19], when co-expressed with other receptors, such as HER2 and HER3, signalling through these receptors promotes proliferation and overrides the effects seen when HER4 is expressed in isolation [20]. However, the exact mechanism of this HER4 mediated effect remains unclear; our data links HER4 to low proliferative indices [2] but HER4 is also linked to apoptosis [13]. Coexpression of other HER family members may, therefore, explain some of the conflicting reports on survival both here and in published studies.

In addition, this cohort of patients is a tamoxifen treated group, the majority of which are ER positive. Previous studies, including ours, have suggested a greater tendency for HER4 to be associated with ER positive tumours [5,21,22]. Within this generally less aggressive set of cancers, the effect of HER4 may be less pronounced.

## Conclusion

We have demonstrated that antibodies against two different HER4 receptor antigen sites identify clear differences in staining patterns. The differences in published reports may well reflect the differing abilities of antibodies to detect distinctly different HER4 functions. The ability of TACE and presenilin to cleave HER4 and modify its activity suggest that careful attention to the subcellular localisation of both cleaved and intact HER4 is essential when investigating its mechanisms of action. It is possible that antibodies more specifically targeting the TACE or BH3 domain may prove valuable in further elucidating the functions of HER4, particularly in regard to impact on clinical outcome in breast cancer.

## Abbreviations

BH3 = BCL-2 homology 3 domain; EGFR = epidermal growth factor receptor; ER = oestrogen receptor; HER = human epidermal growth factor receptor; ICD = intracellular domain; SD = standard deviation; TACE = TNFα-converting enzyme; TMA = tissue microarray.

## Competing interests

The authors declare that they have no competing interests.

## Authors' contributions

ST constructed the TMAs, performed the immunohistochemistry and drafted the manuscript. BD identified representative tumour areas for TMA construction. CW assisted with development of immunohistochemistry protocols. JMS conceived of the study and participated in the design. TC and JMS helped coordinate the study and draft the final manuscript.

## Figures and Tables

**Table 1:** Patient clinical and pathological variables

Variable	Number/total	Valid (%)
Grade		
1	99/391	25.32
2	193/391	49.36
3	99/391	25.32
Unknown	11	
Nodal status		
0	193/369	53.1
1–3	107/369	29.1
4+	69/369	17.8
Unknown	33	
Histological type		
Ductal	322/397	80.1
Lobular	45/397	11.6
Other	30/397	8.3
Unknown	5	
Size		
T1 (<20 mm)	154/380	40.53
T2 (20–50 mm)	204/380	53.68
T3 (>50 mm)	22/380	5.79
Unknown	22	
NPI		
<3.5	128/344	37.21
3.5–5.5	106/344	30.81
5.5+	110/344	31.98
Missing	58	
Age		
<50 years	73/402	36.5
>50 years	328/402	63.5

Grade is the Bloom and Richardson grade. Nodal status is the number of positive nodes. Pathological type: ductal, invasive ductal carcinoma; lobular, invasive lobular carcinoma; other includes mucinous, mucoid and so on. NPI, Nottingham Prognostic Index (grade + nodal status + 0.02 × size in mm).

**Table 2:** Comparison of H4.77.16 and HFR1 staining patterns in the membrane, cytoplasm and nucleus

		H4.77.16			Kappa value
		Negative	Positive	Total	

Membrane					0.426 (*p *< 0.001)
HFR1	Negative	273	28	301	
	Positive	10	18	28	
	Total	283	46	329	
Cytoplasmic					0.351 (*p *< 0.001)
HFR1	Negative	41	15	56	
	Positive	68	205	273	
	Total	109	220	329	
Nuclear					0.051 (*p *< 0.018)
HFR1	Negative	21	3	24	
	Positive	194	111	305	
	Total	215	114	329	

Chi-square where numbers of cases negative or positive for each antibody at the membrane, cytoplasm or nucleus are shown using cut offs defined in the text. Cohen's Kappa value measures the agreement between the two antibodies, where a value of 1 indicates perfect agreement and a value of 0 indicates agreement is no better than chance.

**Table 3:** Membranous HER4: correlation with pathological variables and HER family

	H4.77.16			HFR1		
	χ^2^	*P *value	Correlation	χ^2^	*P *value	Correlation

NPI	4.799	0.091		6.095	0.047	Positive
Size	8.652	0.013	Positive	8.608	0.008	Positive
Grade	2.453	0.293		0.75	0.687	
Nodal	1.769	0.413		3.202	0.202	
EGFR	Fishers	0.019	Positive	Fishers	0.074	
HER2	1.657	0.243		Fishers	0.314	
HER3	53.67	<0.001	Positive	Fishers	<0.001	Positive

Grade is the Bloom and Richardson grade. Nodal status is the number of positive nodes. NPI, Nottingham Prognostic Index (grade + nodal status + 0.02 × size in mm). EGFR, epidermal growth factor receptor.

**Table 4:** Cytoplasmic HER4: correlation with pathological variables and HER family

	Test	H4.77.16 histoscore		HFR1 histoscore	
		*P *value	Correlation	*P *value	Correlation

NPI	Kendall's tau-b	0.004	Positive	<0.001	Positive
Size	Kendall's tau-b	<0.001	Positive	0.021	Positive
Grade	Kendall's tau-b	0.076		<0.001	Positive
Nodal	Kendall's tau-b	0.004	Positive	<0.001	Positive
EGFR	Mann-Whitney	0.035		0.436	
HER2	Mann-Whitney	0.381		0.771	
HER3	Mann-Whitney	<0.001	Positive	0.001	Positive

Grade is the Bloom and Richardson grade. Nodal status is the number of positive nodes. NPI, Nottingham Prognostic Index (grade + nodal status + 0.02 × size in mm). EGFR, epidermal growth factor receptor.

## References

[B1] Witton CJ, Reeves JR, Going JJ, Cooke TG, Bartlett JM (2003). Expression of the HER1-4 family of receptor tyrosine kinases in breast cancer. J Pathol.

[B2] Tovey SM, Witton CJ, Bartlett JM, Stanton PD, Reeves JR, Cooke TG (2004). Outcome and human epidermal growth factor receptor (HER) 1–4 status in invasive breast carcinomas with proliferation indices evaluated by bromodeoxyuridine labelling. Breast Cancer Res.

[B3] Kew TY, Bell JA, Pinder SE, Denley H, Srinivasan R, Gullick WJ, Nicholson RI, Blamey RW, Ellis IO (2000). c-erbB-4 protein expression in human breast cancer. Br J Cancer.

[B4] Srinivasan R, Gillett CE, Barnes DM, Gullick WJ (2000). Nuclear expression of the c-erbB-4/HER-4 growth factor receptor in invasive breast cancers. Cancer Res.

[B5] Pawlowski V, Revillion F, Hebbar M, Hornez L, Peyrat JP (2000). Prognostic value of the type I growth factor receptors in a large series of human primary breast cancers quantified with a real-time reverse transcription-polymerase chain reaction assay. Clin Cancer Res.

[B6] Knowlden JM, Gee JM, Seery LT, Farrow L, Gullick WJ, Ellis IO, Blamey RW, Robertson JF, Nicholson RI (1998). c-erbB3 and c-erbB4 expression is a feature of the endocrine responsive phenotype in clinical breast cancer. Oncogene.

[B7] Suo Z, Risberg B, Kalsson MG, Willman K, Tierens A, Skovlund E, Nesland JM (2002). EGFR family expression in breast carcinomas. c-erbB-2 and c-erbB-4 receptors have different effects on survival. J Pathol.

[B8] Lodge AJ, Anderson JJ, Gullick WJ, Haugk B, Leonard RC, Angus B (2003). Type 1 growth factor receptor expression in node positive breast cancer: adverse prognostic significance of c-erbB-4. J Clin Pathol.

[B9] Abd El-Rehim DM, Pinder SE, Paish CE, Bell JA, Rampaul RS, Blamey RW, Robertson JF, Nicholson RI, Ellis IO (2004). Expression and co-expression of the members of the epidermal growth factor receptor (EGFR) family in invasive breast carcinoma. Br J Cancer.

[B10] Srinivasan R, Poulsom R, Hurst HC, Gullick WJ (1998). Expression of the c-erbB-4/HER4 protein and mRNA in normal human fetal and adult tissues and in a survey of nine solid tumour types. J Pathol.

[B11] Clark DE, Williams CC, Duplessis TT, Moring KL, Notwick AR, Long W, Lane WS, Beuvink I, Hynes NE, Jones FE (2005). ERBB4/HER4 potentiates STAT5A transcriptional activity by regulating novel STAT5A serine phosphorylation events. J Biol Chem.

[B12] Williams CC, Allison JG, Vidal GA, Burow ME, Beckman BS, Marrero L, Jones FE (2004). The ERBB4/HER4 receptor tyrosine kinase regulates gene expression by functioning as a STAT5A nuclear chaperone. J Cell Biol.

[B13] Vidal GA, Naresh A, Marrero L, Jones FE (2005). Presenilin-dependent {gamma}-Secretase Processing Regulates Multiple ERBB4/HER4 Activities. J Biol Chem.

[B14] Lee HJ, Jung KM, Huang YZ, Bennett LB, Lee JS, Mei L, Kim TW (2002). Presenilin-dependent gamma-secretase-like intramembrane cleavage of ErbB4. J Biol Chem.

[B15] Rio C, Buxbaum JD, Peschon JJ, Corfas G (2000). Tumor necrosis factor-alpha-converting enzyme is required for cleavage of erbB4/HER4. J Biol Chem.

[B16] Junttila TT, Sundvall M, Lundin M, Lundin J, Tanner M, Harkonen P, Joensuu H, Isola J, Elenius K (2005). Cleavable ErbB4 isoform in estrogen receptor-regulated growth of breast cancer cells. Cancer Res.

[B17] Tovey SM, Dunne B, Forsyth A, Witton CJ, Cooke TG, Bartlett JM (2005). Can molecular markers predict when to implement treatment with aromatase inhibitors in invasive breast cancer?. Clin Cancer Res.

[B18] Sartor CI, Zhou H, Kozlowska E, Guttridge K, Kawata E, Caskey L, Harrelson J, Hynes N, Ethier S, Calvo B (2001). Her4 mediates ligand-dependent antiproliferative and differentiation responses in human breast cancer cells. Mol Cell Biol.

[B19] Williams EE, Trout LJ, Gallo RM, Pitfield SE, Bryant I, Penington DJ, Riese DJ (2003). A constitutively active ErbB4 mutant inhibits drug-resistant colony formation by the DU-145 and PC-3 human prostate tumor cell lines. Cancer Lett.

[B20] Yarden Y (2001). The EGFR family and its ligands in human cancer. signalling mechanisms and therapeutic opportunities. Eur J Cancer.

[B21] Tang CK, Concepcion XZ, Milan M, Gong X, Montgomery E, Lippman ME (1999). Ribozyme-mediated down-regulation of ErbB-4 in estrogen receptor-positive breast cancer cells inhibits proliferation both *in vitro* and *in vivo*. Cancer Res.

[B22] Wright C, Nicholson S, Angus B, Sainsbury JR, Farndon J, Cairns J, Harris AL, Horne CH (1992). Relationship between c-erbB-2 protein product expression and response to endocrine therapy in advanced breast cancer. Br J Cancer.

